# Knowing when digital adds value to health: a framework for the economic evaluation of digital health interventions

**DOI:** 10.1093/oodh/oqae028

**Published:** 2024-12-02

**Authors:** Thomas Wilkinson, Mengxiao Wang, Jed Friedman, Yai-Ellen Gaye, Marelize Görgens

**Affiliations:** Health, Nutrition and Population Global Practice, World Bank, 1818 H Street NW, Washington DC 20433, USA; Health, Nutrition and Population Global Practice, World Bank, 1818 H Street NW, Washington DC 20433, USA; Development Economics Research Group, World Bank, 1818 H Street NW, Washington DC 20433, USA; Health, Nutrition and Population Global Practice, World Bank, 1818 H Street NW, Washington DC 20433, USA; Health, Nutrition and Population Global Practice, World Bank, 1818 H Street NW, Washington DC 20433, USA

**Keywords:** digital health intervention, economic evaluation, low- and middle- income countries, value, health technology assessment, AI

## Abstract

Digital health interventions (DHIs) hold significant promise for addressing health system challenges and the ‘DHI pilot’ is ubiquitous in developing-country contexts. Because the opportunity cost of investing in DHIs can be large, countries must make choices about which interventions to scale up. To make good investment decisions about DHIs, there is a need to define and establish their value within the local health system. Economic evaluation enables a systematic and evidence-based approach to describing value; however, guidance and applied economic evaluation of DHIs in developing country settings are limited. The implementation context and regulatory framework for DHIs in many resource-constrained settings is often fragmented and uncertain, creating unique challenges for economic evaluation. However, limited resources reinforce the need to adopt analytical approaches to manage this uncertainty and inform high-value investments in digital health.

This paper develops an economic evaluation framework to assist in establishing the economic value of DHIs to inform policy, programming and appropriate scale-up in resource-constrained settings. It is intended for country governments and those providing technical assistance in global development related to digital health. The DHI economic evaluation framework consists of 5 steps: (1) determine the context, (2) determine the intervention type, (3) establish the level of complexity, (4) apply the analytic principles and (5) represent the value proposition. The framework facilitates methodological transparency and structure, thereby improving the overall usefulness of economic evaluations of DHIs and a starting point for more comprehensive and localized processes.

**RESUMEN:**

Las Intervenciones de Salud Digital (ISD) ofrecen una promesa significativa para abordar desafíos del sistema de salud y el ‘estudio piloto de ISD’ es ubicuo en el contexto de los países en vías de desarrollo. Dado que el coste de oportunidad de invertir en ISD puede ser alto, los países tienen que tomar decisiones al escoger qué intervenciones escalar. Para tomar buenas decisiones en el financiamiento de las ISD, se necesita definir y establecer su valor dentro del sistema de salud local. La evaluación económica permite adscribir valía de manera sistemática y basándose en pruebas, pero la orientación y evaluación económica aplicada a ISD en países en desarrollo son escasas. El contexto para la implementación y los marcos normativos que operan sobre las ISD suelen ser inciertos y fragmentarios en lugares de limitados recursos, lo que crea desafíos singulares para la evaluación económica. A pesar de lo anterior, el hecho mismo de que los recursos sean limitados subraya la necesidad de adoptar enfoques analíticos para manejar esta incertidumbre e informar la inversión de alto nivel en salud digital.

Este escrito desarrolla un marco de evaluación económica que ayude a establecer el valor económico de las ISD para informar políticas, programación, y escalamiento apropiado, en entornos de recursos limitados. Está dirigido a gobiernos de estado y a quienes proveen asistencia técnica en desarrollo global con relación a salud digital. El marco de evaluación económica de ISD consta de 5 pasos: (1) determina el contexto; (2) determina el tipo de intervención; (3) establece el nivel de complejidad; (4) aplica los principios analíticos; y (5) representa la propuesta de valor. El Marco facilita la transparencia y estructura metodológicas, mejorando así la utilidad general de las evaluaciones económicas de las ISD y brindando un punto de partida para procesos más exhaustivos y localizados.

**RESUMO:**

As intervenções de saúde digitais (DHI) são muito promissoras para enfrentar os desafios do sistema de saúde e o ‘piloto DHI’ é omnipresente nos contextos dos países em desenvolvimento. Uma vez que o custo de oportunidade do investimento em IDS pode ser elevado, os países têm de fazer escolhas sobre quais as intervenções a alargar. Para tomar boas decisões de investimento nas IDS, é necessário definir e estabelecer o seu valor no âmbito do sistema de saúde local. A avaliação económica permite uma abordagem sistemática e baseada em provas para descrever o valor. No entanto, as orientações e a avaliação económica aplicada das IDS nos países em desenvolvimento são limitadas. O contexto de implementação e o quadro regulamentar das IDS em muitos contextos com recursos limitados são frequentemente fragmentados e incertos, criando desafios únicos para a avaliação económica. No entanto, os recursos limitados reforçam a necessidade de adotar abordagens analíticas para gerir esta incerteza e informar os investimentos de elevado valor na saúde digital.

Este documento desenvolve um quadro de avaliação económica para ajudar a estabelecer o valor económico das DHI para informar a política, a programação e a expansão adequada em contextos de recursos limitados. Destina-se aos governos nacionais e aos que prestam assistência técnica no desenvolvimento global relacionado com a saúde digital. O quadro de avaliação económica das IDS é composto por 5 etapas: (1) determinar o contexto, (2) determinar o tipo de intervenção, (3) estabelecer o nível de complexidade, (4) aplicar os princípios analíticos e (5) representar a proposta de valor. O Quadro facilita a transparência e a estrutura metodológica, melhorando assim a utilidade global das avaliações económicas das IDS e constituindo um ponto de partida para processos mais abrangentes e localizados.

**RÉSUMÉ:**

Les interventions de santé numérique (ISN) sont très prometteuses pour relever les défis du système de santé et le « projet pilote ISN » est omniprésent dans les contextes des pays en développement. Étant donné que le coût de l’opportunité d’investissement dans les ISN peut être important, les pays doivent faire des choix quant aux interventions à intensifier. Pour prendre de bonnes décisions d’investissement concernant les ISN, il est nécessaire de définir et d’établir leur valeur au sein du système de santé local. Une évaluation économique permet une approche systématique et fondée sur des données probantes pour décrire leur valeur, mais les directives et l’évaluation économique appliquée des ISN dans les pays en développement sont limitées. Le contexte de mise en œuvre et le cadre réglementaire des ISN dans de nombreux contextes aux ressources limitées sont souvent fragmentés et incertains, créant des défis uniques pour l’évaluation économique. Cependant, les ressources limitées renforcent la nécessité d’adopter des approches analytiques pour gérer cette incertitude et éclairer les investissements à forte valeur ajoutée dans la santé numérique.

Ce document développe un cadre d’évaluation économique pour aider à établir la valeur économique des ISN afin d’éclairer les politiques, la programmation et une mise à l’échelle appropriée dans des contextes aux ressources limitées. Il est destiné aux gouvernements des pays et à ceux qui fournissent une assistance technique dans le développement mondial lié à la santé numérique. Le cadre d’évaluation économique des ISN comprend 5 étapes: (1) déterminer le contexte, (2) déterminer le type d’intervention, (3) établir le niveau de complexité, (4) appliquer les principes analytiques et (5) représenter la proposition de valeur.. Le cadre facilite la transparence et la structure méthodologiques, améliorant ainsi l’utilité globale des évaluations économiques des ISN et constituant un point de départ pour des processus plus complets et localisés.

## INTRODUCTION

The use of digital health interventions (DHIs) in both developed and developing country contexts is growing rapidly, with the promise of benefits to health systems and the people who use them. While DHIs can improve health, wellness and health system efficiency, the cost can be substantial and often require irretrievable upfront investments. Governments have to prioritize which DHIs to scale. One of the most effective tools available to generate information for prioritization is economic evaluation—the comparative analysis of one or more interventions in terms of costs and consequences [1].

In many developing countries, there are substantive barriers to conducting and using analysis to inform resource allocation and investment in health interventions and programs [2]. This may be more pronounced for DHIs where there are weak or insufficient regulatory frameworks, fragmented information systems, and heterogeneous procurement systems. This complex digital health ecosystem, coupled with often acute health funding shortages, makes it even more essential and timely for a DHI economic evaluation framework to be introduced and utilized. The scarcity of resources for health means that the opportunity cost of a poor value investment can have extensive negative population health consequences. Digital health has already arrived in many developing countries, and the ‘DHI pilot’ is a common occurrence. Targeted advocacy and vertical disease programs mean that attempts at implementation and scale up of DHIs will occur through domestic or development funding whether economic evaluation is used within decision making or not. The challenge therefore is how to foster progress towards using economic evaluation for DHIs in developing country contexts to encourage and support high value DHI investments, and to limit poor value DHI investments.

The World Bank and the International Institute for Impact Evaluation developed an evidence gap map (EGM) to investigate the available published literature on impact evaluations of DHIs [3]. The EGM involved a comprehensive assessment of available evidence and revealed a limited economic evaluation evidence base for DHIs, particularly in LMIC context. This dearth of evidence is likely to have multiple causes, including research funding, technical capacity, and demand from decision makers. However, one cause of the limited economic evidence is expected to be the limited available guidance to motivate and inform economic evaluation.

The DHI Economic Evaluation Framework (the framework) has been developed by the World Bank in consultation and collaboration with an extensive stakeholder network of practitioners and users of economic evaluation, and those involved in digital health in developing country contexts. It aims to support countries to analytically represent the value of DHIs in context while adhering to the central principles of economic evaluation. It attempts to achieve a balance between prescriptive standards for economic evaluation, and more nuanced principles to empower analysts and decision makers to conduct and use economic evaluation of DHIs more effectively. The framework is an enabler of recommendations of the World Bank’s Digital-in-Health report, which called for countries to unlock the value of digital health by taking steps to ‘prioritize, connect, and scale’. Specifically, the framework supports the production of evidence for priority setting, and moving from pilot to wider implementation and scaling. Further material relating to digital health and the framework is being conducted by the World Bank and this paper provides an outline of an approach to economic evaluation of DHIs and a direction of travel for work in this area.

The framework utilizes the World Health Organization’s (WHO) classification of DHIs [4], with additional consideration for economic evaluation of DHIs that are enabled by predictive analytics and non-DHIs as part of a wholistic intervention. The framework is also intended to be used within the larger guidance and technical support ecosystem for investments in digital health, represented in the *Digital Implementation Investment Guide* (DIIG) [5], a global initiative coordinated by WHO to outline the steps for country investment in digital health.

## SCOPE: DHIS AND ARTIFICIAL INTELLIGENCE

This framework applies to the broad and diverse grouping of DHIs. WHO adopts a user-focused categorization of DHIs, specifying DHIs that are predominantly used by:

1. Persons or Individuals, who receive, e.g. targeted text message alerts or medication reminders;
2. Health-care providers, who receive, e.g. clinical decision support or diagnostic assistive interventions;
3. Health management and support personnel, who receive, e.g. digital interventions to monitor or organize human resources;
4. Data services, which receive, e.g. digital interventions for data synthesis and visualization or to parse unstructured data into structured data

In addition to a user-focused classification, Services and Application types are categorized into:

a) Data management services
b) Surveillance and Response

**Figure 1:**
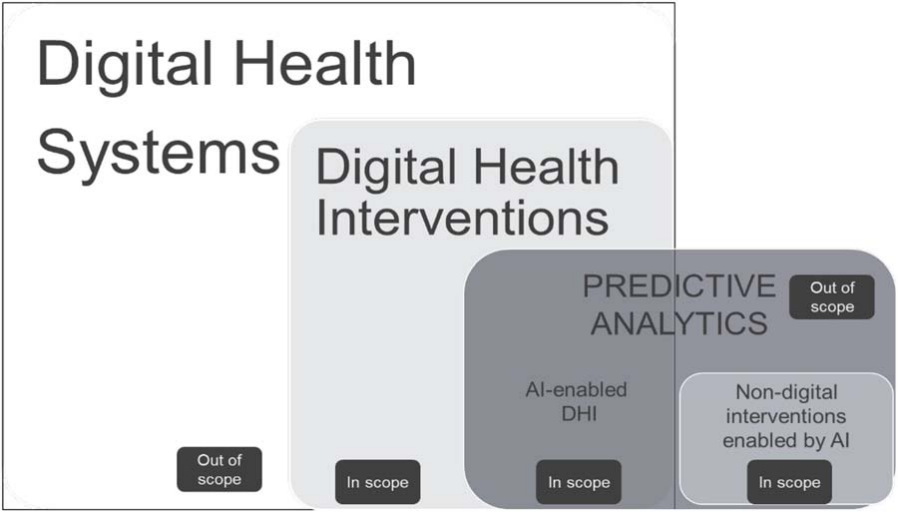
Representation of the scope of the DHI economic evaluation framework

The advancement of digital health and the large volume of data being generated through digitizing health information and development in mobile health applications has promoted artificial Intelligence (AI) as an enabler of health in a digitized world [6]. To meet the range of available DHIs, the scope of this framework also incorporates technologies enabled by AI processes and systems but excludes predictive analytic applications that are not incorporated within a health intervention (Figure 1). The categories of AI applications [6] include:

- Machine learning
- Natural language processing
- Automated planning and scheduling (or AI planning)
- Image and signal processing

As the digital health and AI field is constantly evolving, these are not intended to be an exhaustive categorization but a representation of common types of AI that may be feasible candidates for economic evaluation.

## THE CASE FOR A DHI ECONOMIC EVALUATION FRAMEWORK

### Methods frameworks for economic evaluation in health

A methods framework guides the planning, conduct and reporting of economic evaluation and assists in maintaining a consistent approach allowing comparison of analytical results over time and context. An economic evaluation framework builds on the concept of a reference case but is less prescriptive and accommodates a wider range of methodological options.

The World Bank has published substantively in applied benefit–cost analysis with methodological recommendations developed as early as the 1970s, with subsequent methods initiatives in 1992 and 2010 [7]. The concept of a reference case within a framework was first used by the first US panel of cost and cost effectiveness in health care in 1996 [8]. The 2000s saw a proliferation of national priority-setting agencies, such as the National Institute for Health and Care Excellence in UK and the Health Intervention and Technology Assessment Program in Thailand, that built on the concept of specified methods for economic evaluation to inform investment decisions for local national health systems. While these initiatives are a significant stimulus for economic evaluation methods innovation, the direct global application of these frameworks is limited, as by definition these are developed from the perspective of a local decision maker concerned with national budgets and national population health in a particular country.

A methods framework can be generalized or intervention or disease specific. Generalized frameworks are typically adopted by national health technology assessment agencies or groups for a specific jurisdiction and apply to any type of health intervention or technology in any therapeutic area. For example, the EUnetHTA Core Model is a comprehensive methodological framework developed by European Union member states for collaborative production and sharing of health technology assessment information [9]. An intervention- or disease-specific framework attempts to provide more targeted guidance related to a particular group of interventions or therapeutic areas, under an assumption that more explicit and specific guidance is required, e.g. to assess interventions in oncology or infectious disease. Importantly, there is no universally correct framework to apply, and differing frameworks may complement each other depending on context, nature of the intervention to be assessed and the needs of the decision maker who is the intended recipient of the analysis.

### DHIs and the concept of value

**Figure 2:**
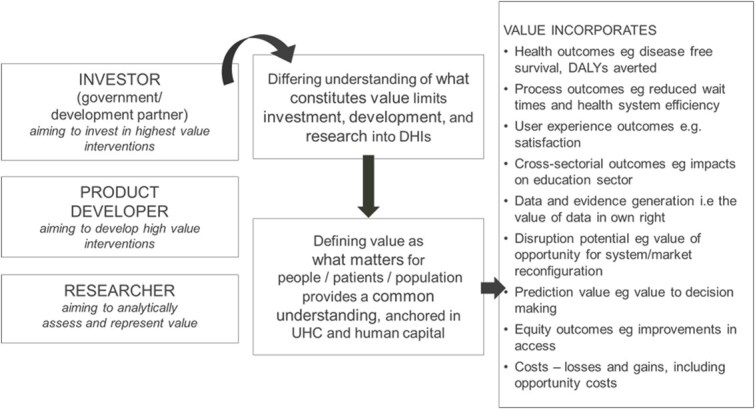
The concept of value in an economic evaluation. *Note:* DALY = disability adjusted life year, a composite measure of burden of disease incorporating morbidity and mortality; UHC = universal health coverage

The concept of value in health and health care is highly contested in the literature, with an abundance of value conceptualizations that seek to accurately reflect the ‘things that matter’ to payers, patients and the population impacted by the investment or implementation of a health intervention [10]. While approaches to value conceptualization necessarily change over time with emerging research and developments in the field, a consistent approach to decision making and economic evaluation requires a consistent approach to the representation of value. Figure 2 depicts major areas of value that are expected to be held in common, to a greater or lesser degree, by the three main audiences or potential users of economic evaluation of DHIs: investors, product developers and researchers. These value areas are also informed by the findings of the DHI EGM [11]. Effective decision-making processes require economic evaluation that can appropriately reflect value, and therefore any analytical methodology should be tailored to the conceptualization of value related to DHIs.

### Existing guidance on the evaluation of DHIs

WHO’s guide to *Monitoring and Evaluating Digital Health Interventions* provides a strong foundation for the assessment and generation of evidence on DHIs [12]. Its main focus, however, is to describe methods for monitoring and evaluation of DHIs rather than to provide extensive guidance on DHI economic evaluation.

A systematic review noted that a central challenge in the value assessment of DHIs was due to the complexity of the simultaneous evaluation of clinical, organizational and economic aspects [13].

The review proposed several recommendations regarding the criteria to be considered in the measurement of value of DHIs, as detailed in Table 1.

**Table 1:** Summary findings from review of existing DHI analytical frameworks

FIVE RECOMMENDATIONS	
**Choice of comparator**	The value of a DHI should be determined by the incremental advantage compared to the current standard of care.
**Multistakeholder perspective**	The value of a DHI should quantify incremental differences it delivers to all beneficiaries.
**Organizational impact**	The value of DHIs should be conditional on the health-care system preparedness to absorb efficiency gains and assurance that data generated by DHI will be accessible to health-care professionals.
**Multidimensional outcomes**	The value delivered by digital health solutions should consider multiple dimensions such as clinical, organizational, behavioral and technical.
**Interoperability**	Connectivity to other data sources must be considered in the evaluation of a digital health solution.
**ONE METHODOLOGICAL SUGGESTION**	
**Value aggregation function**	Given the multiple criteria to be considered in the pricing and reimbursement process, each value attribute should be weighted in any calculations based on the preferences of chosen stakeholder groups, and the aggregate value score for each DHI should be estimated.

*Source:* Adapted from Kolasa et al. [12].

A methodological systematic review [14] investigated published analytical frameworks and outcome measures specifically related to digital health. The review highlighted the unique dimensions of DHI economic evaluation including the finding that traditional frameworks (specifying cost effectiveness analysis, cost utility analysis, cost benefit analysis) and outcome measures may not appropriately determine the full value proposition of DHIs. The review found multiple frameworks targeting specific areas within digital health, such as telemedicine, and did not identify any frameworks with an intended focus on assessments in a developing country context. The review identified an influential paper that proposed a series of research questions that assist in designing analysis that is tailored to generating evidence for decision making related to DHIs [15].

Within a developed country context, national health technology assessment agencies are progressing analytical methods and processes for the unique assessment of DHIs. For example, the UK’s National Institute for Health and Care Excellence has developed an evidence standards framework that details the approach to the use of evidence when an evaluation is conducted to inform investments in the National Health Service in England and Wales [16]. The Finnish national HTA coordination unit FinCCHTA has developed ‘Digi-HTA’ as a specific method and process for assessment of digital products and services for use in the Finish health system.

A review of generalized eHealth policy frameworks in low and lower-middle-income country (LLMICs) [17] found significant gaps in eHealth policy development, indicating that many developing countries are not yet in positions to develop economic frameworks to inform decision making as part of established policy. The ideal scenario for methods framework development is country-ownership and production, but the need for evaluation frameworks in developing country contexts exists regardless of local capacity and system challenges. In line with nascent health technology assessment systems in LLMIC settings, we are not aware of current established DHI economic evaluation frameworks adopted by government bodies in developing country contexts. Digital technologies, by their global nature, will enter health systems at all levels of development, making the need for robust value assessment of DHIs in developing country context more acute.

### DHIs and the role of evidence

DHIs are unique among health interventions in the way that evidence is generated and synthesized. An economic evaluation typically views the range of evidence for a health intervention at a single point in time, treating an intervention as static with established efficacy when used at a set dose or in a particular way, in a defined patient group. The effectiveness of DHIs is highly variable, and the performance and composition of a DHI can adapt throughout the lifecycle.

DHIs, in common with many other complex interventions, are intricately integrated into the context within which they are used. This diminishes the importance of methods relying on established efficacy and limits the applicability of the traditional evidence hierarchy. The randomized controlled trial (RCT) remains a gold standard for establishing treatment effect; however, RCTs in isolation are unlikely to be able to provide the intervention effectiveness estimate required in an economic evaluation of a DHI. In developing countries that have historically needed to transfer evidence from RCTs conducted in different contexts, this reinforces the need to seek localized and context specific evidence to assess DHIs. However, advances in reporting standards for trials of DHIs have improved quality and utility of trial data. The SPIRIT-AI and CONSORT-AI statements provide clear specification for the design and reporting of protocols and trials of interventions involving AI components [18, 19]. They expand on the established CONSORT and SPIRIT statements with specific components to enable the generation of evidence related to AI technologies.

Poor and fragmented information systems often pose a barrier to the generation and use of real-world evidence for economic evaluation. A DHI, however, often generates data through its use. The data gathered will inform basic parameters such as utilization, but may also enable links with other sources to impact on health and processes dynamically. As many DHIs include features of adaptive design, the generation of evidence can lead directly to changes in the effect size of the intervention. There is also substantive scope for DHIs to dynamically improve the effectiveness of other (digital and nondigital) interventions as a result of improved evidence; in this way, the evidence supporting DHIs may not be a direct clinical effect of the intervention, but of the changes to existing care that may be more efficient or impactful. Considerations for the assessment of digital predictive capabilities can build on existing economic evaluation of nondigital risk-prediction models.

The regulatory environment for a particular type of health intervention is a major driver of the approach to evidence generation. The strong traditional focus of health intervention regulation on patient safety and efficacy means that evidence generation is commonly focused on whether potential benefits outweigh potential harms rather than cost effectiveness or value for money. When conducting economic evaluation for an intervention that already has regulatory approval (such as pharmaceutical interventions), evidence required at the point of regulation will have demonstrated efficacy and safety for a particular clinical indication. In contrast, economic evaluations of nonregulated products (or for products in contexts where the regulatory environment does not routinely require demonstrated efficacy through clinical trial) may not have baseline evidence of impact to parameterize an analysis.

The regulatory environment for health interventions in many resource-constrained settings is often characterized by limited coordination and weak specification and enforcement. While some global initiatives such as the World Health Organization’s Global Model Regulatory Framework for Medical devices [20] provides a basis for general device regulation, some digital interventions may be used in country without any formal regulatory requirements, while others may align to different specifications depending on country of development or prior market authorization. This creates specific challenges for economic evaluation and establishes the need for a framework that includes requirements relating to intervention specification and characterization.

Despite the dynamics of evidence generation and use for DHIs that are clearly different from traditional health interventions, DHIs should not be seen as exceptional or warranting a lower standard of evidence to inform economic evaluation or decision making [16].

## THE DHI ECONOMIC EVALUATION FRAMEWORK

### The DHIs economic evaluation framework

**Figure 3:**
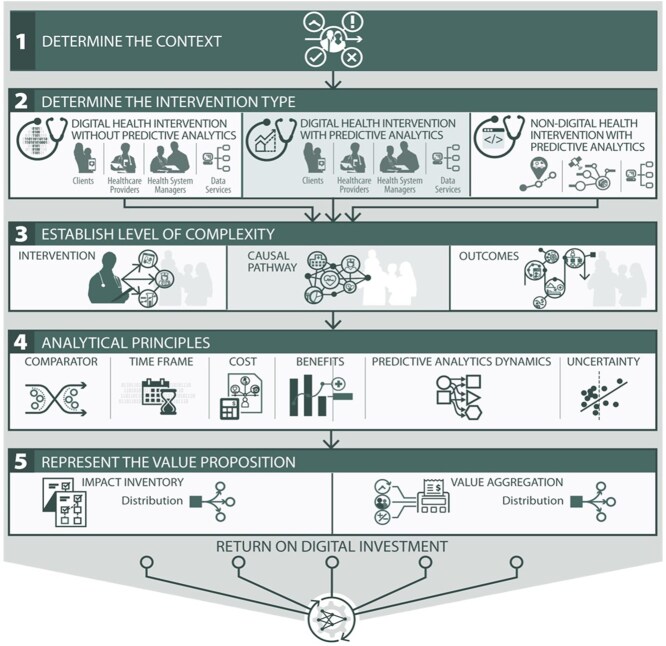
Digital health intervention economic evaluation framework

This section describes the DHI Economic Evaluation Framework, outlining the main elements, principles and specifications as detailed in Fig. 3.

**STEP 1: identify and represent the context of the economic evaluation**

The first step in an economic evaluation of a DHI is to determine the context in which the intervention is to be assessed. A DHI is a context-specific intervention, with costs and effects highly contingent on the health system and digital architecture in which it is implemented. When determining context, it is important to specify general contextual elements, including the health system characteristics, how the intervention would be used in the clinical or treatment pathway, and user type. The digital context of the intervention is also an essential element and includes digital architecture and equipment, information systems and software where applicable. In a developing country setting, the context might be highly heterogeneous limiting the comprehensive definition of context. Analysts should attempt to describe the context with as much detail as possible given existing constraints, using available tools, such as the Digital Health Investment Review Tool developed under the Maternal and Child Survival Program [21].

The intended decision maker is the organization, body or group of people who will make use of the information produced in the economic evaluation. It is an important component of the economic evaluation, as it informs interpretation and application of all other methodological concepts, including the perspective of the analysis.

Intrinsically linked to the intended decision maker is the context in which the DHI will be implemented. The context determines multiple aspects of the evaluation including DHI functionality, use, impact and costs. The context of the DHI should therefore be established from the outset, with clear explanations of key components of the context, including the country context, health system environment and digital enabling environment.

#### STEP 2: determine the intervention type

**Figure 4:**
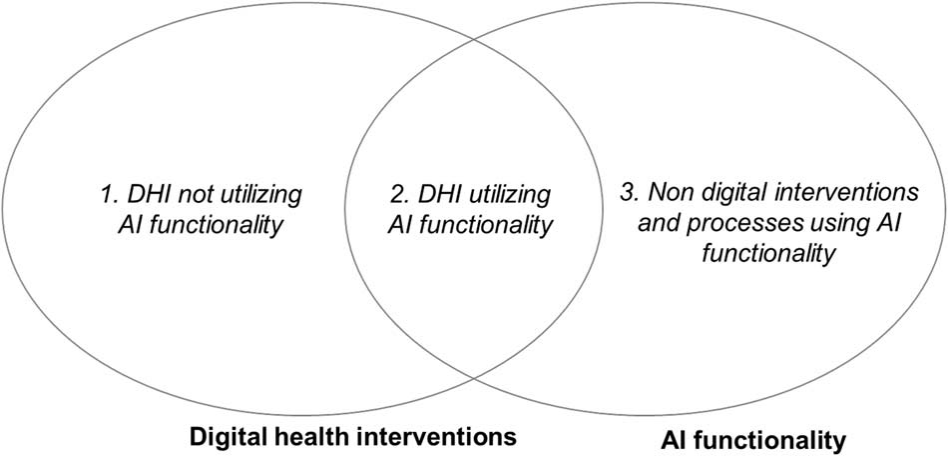
Health interventions with and without AI

Specification of the intervention, including its use-case and intended population, is an essential element of the evaluation. This framework requires not only a general description of the intervention but also alignment to established classification systems. In the first instance, the intervention should be assigned to a WHO DHI type, with specification as to whether there is a predictive analytics component. This creates three major categories of DHI, with further granularity for DHIs under WHO’s classification system, as illustrated in Fig. 4.

1. DHIs defined under WHO’s DHI classification system without AI-enabling functionality, e.g. simple text message to patients as a reminder for regular follow-up.
2. DHIs defined under WHO’s DHI classification system with AI-enabling functionality, e.g. machine learning radiology diagnostics.
3. Non-DHIs enabled by the use of AI technology, e.g. use of demographic and clinical information for identification of at-risk patients for health professional consultation.

#### STEP 3: establish the level of complexity

The third step in the framework is to establish the level of complexity, clearly identifying key elements to inform the approach in Steps 4 and 5. The concept of intervention complexity and implications for evaluation are not unique to DHIs and have been well established in the literature, particularly in the areas of program evaluation and public health intervention [22].

In a discussion paper on economic evaluation of DHIs, the critical importance of reflecting DHIs as complex interventions within complex systems was highlighted [23]. The paper noted the description of a complex intervention by the Medical Research Council and National Institute for Health Research [24, 25] as one that ‘contains several interacting components, and other characteristics, such as the number and difficulty of behaviors required by those delivering or receiving the intervention.’ The proposed approach to representing complexity in the framework is detailed in Table 2 and builds on the approach by Petticrew et al. [26] and highlighted by McNamee et al. [23].

**Table 2:** Digital health interventions and complexity

TYPE	DESCRIPTION
**Intervention complexity**	Many DHIs are multi-attribute, with interdependent parts and delivery mechanisms, and often adapt over time.
**Causal pathway complexity**	The causal pathway linking observed outputs to outcome and impact can have substantial complexity, compounded by range of identified outcomes and complex attribution and feedback loops.
**Outcome complexity**	DHIs commonly have a range of outcomes, including health and health system effects, and involve spillovers and externalities, impact on patients, users and effects on the wider population.

*Source:* Petticrew et al. [25]; McNamee [22].

The approach requires specifying three types of complexity. *Intervention complexity* relates to the components of the intervention itself. DHIs commonly have multiple parts, which combine to produce the observed effect. Intervention complexity is compounded by the continual adaption and modification of DHIs over time. An example highly complex intervention is the HIV Infant Tracking System (HITSystem) a web-based tool for management of care of HIV-exposed infants in Kenya. The HITSystem comprised multiple components, including messaging, alerts, reporting and care coordination all intended to improve testing and anti-retroviral initiation and patient retention [27]. *Causal pathway complexity* refers to linking attribution of an impact to the initial input. Frequently the realization of the impact of a DHI involves multiple moderators and this complexity is also highly linked to implementation context. Many of the interventions identified in a review of DHIs in the management of hypertension in LMIC settings represented causal pathway complexity; where the interventions would establish monitoring and feedback of information, which in turn would aim to improve adherence to medication and lifestyle choices, leading to reduced blood pressure and lower risk of adverse cardiovascular outcomes [28]. *Outcome complexity* refers to the complexity associated with determining impact, as DHIs may have externalities and spillovers. A review of mobile phone-based digital interventions to improve community health worker performance in developing countries identified outcomes including productivity, improved reporting, better patient management and satisfaction and knowledge [29]. These often relatively simple interventions, (such as routine text-messaging reminders) had multiple complex outcomes that require identification for economic evaluation to be conducted. While the particular approach to representing the level of complexity of the DHI will likely depend on the requirements of the analysis, users should at least explicitly acknowledge the components of complexity and use it to inform the methodological choice in step 4.

#### STEP 4: apply analytical principles

The fourth step of the framework is to apply economic evaluation principles as detailed in Table 3. This builds on the extensive methods literature in economic evaluation [1–30]. It is beyond the scope of this framework document to replicate existing methodological guidance, but the framework is used to identify the principles that should ideally be followed when making context- and intervention-specific methods choices. An important consideration is that while there may be some variation to the particular approach of any economic evaluation, these principles are expected to apply for both digital and nondigital interventions (with the exception of predictive analytics that are expected to be unique to DHIs with a predictive analytics component). The principles below present challenges in developing countries where the data and information systems are not able to accurately track health outcomes, and services may be provided free at the point of use, limiting available cost information. The over-arching objective however is that an attempt should be made to address the relevant principle—i.e. to gather or estimate costs and health outcomes in the best way possible, acknowledging that even in developed countries, no economic evaluation is conducted using perfect information.

**Table 3:** Analytical principles

ITEM	PRINCIPLE
**Comparator**	The comparator(s) against which costs and benefits are measured should accurately reflect the decision problem.
**Time frame**	The time horizon of the analysis should be of sufficient length to capture all significant differences between the intervention and comparator.
**Costs**	All differences between the intervention and the comparator in expected resource and costs of delivery to the target population(s) should be incorporated into the evaluation.
**Benefits**	All differences between the intervention and the comparator in expected benefits to the target population(s) should be incorporated into the evaluation.
**Predictive analytics**	The dynamics of predictive analytics on evidence generation, costs and benefits should be reflected in the analysis.
**Uncertainty**	The uncertainty associated with the evaluation estimates should be appropriately represented.

Establishing the comparator against which the intervention will be evaluated is fundamental to the approach and outputs of an analysis. Economic evaluation seeks to represent costs and consequences in context, which requires understanding of what the intervention would replace, including whether the current practice is doing nothing or taking minimal action. However, it is often difficult to determine exactly what would be replaced by an intervention, and so this framework recommends the development of two comparator scenarios, drawing on the approach of the International Decision Support Initiative (IDSI)’s reference case [31]. Analyst judgment is required to determine the validity of each scenario depending on the context and needs of the analysis. The first scenario is standard of care or existing process, and the second scenario is minimal supportive care. Assessments of DHIs may present additional challenges in identification of the comparator, where the “intervention replaced” may be digital or non-digital, and in many cases multi-modal, e.g. the comparator to a maternal care app may be clinic visits and care provided by a community health worker.

The time frame [31] adopted in an analysis is an important element that ensures that any bias based on the timing of costs and benefits is minimized. The general principle is that the time frame should be of sufficient length to capture all relevant costs and benefits between the intervention and comparator. Where there are observed mortality differences between the intervention and comparator, this will usually require that a lifetime time horizon is adopted. This is particularly relevant for DHIs where a significant proportion of costs are frequently incurred up-front at the stage of development or implementation, with benefits extending into the future. An important consideration for time-frame choice is the discount applied to future costs and effects. This framework does not prescribe a particular annual discount rate but requires that the discount rate chosen is clearly specified and incorporates local contextual evidence.

The framework requires a comprehensive approach to costing, with the principle specifying that ‘all differences between the intervention and the comparator in expected resource and costs of delivery to the target population(s) should be incorporated into the evaluation.’ The approach to collection, synthesis and inclusion of costs in an economic evaluation is detailed extensively in the literature. However, the nature of costing for DHIs is likely to require specific approaches, and it is expected that dedicated research relating to DHI costing is required. In particular, assessment of a DHI is likely to require consideration of how fixed costs are to be attributed in relation to costs of development and future cost structures and at different levels of scale. A ‘legacy’ intervention may pass costs or cost savings onto the health system over a long time period and the marginal cost per user may be substantially reduced with many concurrent DHIs.

In common with costing, estimation of the benefits associated with a DHI should take a comprehensive approach, with the principle that all differences between the intervention and the comparator in expected benefits to the target population(s) should be incorporated into the evaluation. The principle requires in the first instance that benefits are identified in natural units, with approaches to aggregation and synthesis considered in Step 5. The impact inventory details specific areas of value that will guide the organization of the benefits into meaningful categories, aiding interpretation by decision makers. A particular consideration for representing benefits of DHIs is the potential for intervention modification after initial implementation (see Section 3.5 on the role of evidence) [32].

The predictive analytics principle requires that the impact associated with the ability of a DHI to assist in prediction within the anatomy of a task is explicitly detailed and quantified where possible. This category is unique to DHIs with predictive functionality and enables the impact of the prediction component of an intervention to be isolated from the wider impact of the intervention. For example, a radiology diagnostic intervention with predictive analytics may be used for assisting in the diagnosis of tuberculosis disease from radiology scans. The complete diagnostic intervention may have many components, such as digital information capture, automated messaging and referral functionalities, but the prediction value would be represented by the improved diagnostic accuracy in terms of sensitivity and specificity of the intervention compared to either standard of care or combinations of the intervention in conjunction with radiologist prediction and judgment. Representing the prediction value in isolation enables the comparison of differences in prediction value between interventions at different time points and contexts.

This principle interacts with other principles (particularly costs and benefits). However, the unique predictive impacts should be explicitly established in the analysis. The approach to representing predictive analytics dynamics will need to be tailored to the nature of the intervention, with specific consideration given to the identification of the impacts relating to the nature of evidence generation and the disruptive and process changes that may be enabled by the intervention.

Methods for representation of uncertainty in economic evaluation are well established, and analysis should reflect major aspects of uncertainty including parameter, structural and methodological uncertainty [1], incorporating the unique attributes of DHI in relation to evidence generation and use (Section 2).

#### STEP 5: Represent the value proposition

The fifth step in the framework is to represent the value proposition. The framework recommends that the value proposition is explicitly acknowledged and represented both in disaggregated form as an impact inventory (Table 4), and aggregation in a form required by the intended decision maker. Throughout the representation of the value proposition, it is important that the uncertainty and distribution of impacts are clearly represented. While the methodological approaches to representing distribution of impacts is likely to be common across digital and nondigital interventions [33, 34], DHIs have important and unique distributional considerations. First, in many instances DHIs have the potential to mitigate existing access barriers to traditional health-care provision, particularly for marginalized or rural populations. However, there is also potential for DHIs to exacerbate existing inequalities where access to a DHI is limited to those that already have access to an enabling digital health infrastructure, such as a smartphone or a local clinic with an internet connection. While an economic evaluation may not be able to quantify the full distributional impacts of a DHI investment, the value proposition step should attempt to represent impacts using established economic evaluation methods.

**Table 4:** Components of impact inventory

COMPONENT	DESCRIPTION
Health impact	Health impacts: natural units, generalized units and net health benefits that incorporate the opportunity cost of lost health
Health system impact	The impact on the health system as a result of intervention implementation, including direct measurable impacts and longer term predicted process changes or disruption potential
User-experience impact	Impacts on patients, population and health workforce not represented within health or health system impact such as trust, privacy, choice, satisfaction and knowledge
Value of data	The value represented by the availability of data gathered through the use of the DHI that is not represented in direct impacts on health, the health system or user experience
Cross-sectoral impact (beyond health system)	Impacts on the non-health sectors, e.g. education and wider societal benefits, such as human capital

### Disaggregation: impact inventory

The impact inventory specifies five broad inventory areas Table 4, and it is expected that depending on the nature of the DHI being evaluated, additional subcategories will be required. Application of the impact inventory will facilitate further specification and detail on the approach for comprehensive representation of appropriate subcategories, building on existing methodological guidance on outcomes measurement of general health interventions. Identification of the ‘type’ of DHI in Step 2 will guide a generalized approach to representing components of the impact inventory.

An essential element of the impact inventory is that outcomes are represented in units that are applicable to the particular category. DHIs commonly have a mix of outcomes, including those that are health-related and non-health, while others (such as those used by health system managers for operational or organizational tasks) may not have direct or meaningful health impacts at all. The impact inventory is a way to capture these differing benefits. It is likely that the different categories may not be completely mutually exclusive, and some categories will have downstream attribution effects on summary metrics and costs.

The health impact component requires representation of the observed health effects relative to the identified comparator(s), with a requirement for both positive and negative health effects to be represented. The health system impact requires representation of the changes in processes that are expected to be facilitated by the introduction of the DHI and is likely to incorporate intermediate outcomes such as user experience and acceptability, where these effects lead to meaningful process change.

The user-experience impact will commonly be reported in a qualitative synthesis or through use of established tools for measurement of the user experience. It is beyond the scope of this framework to recommend specific user-experience measurement tools. However, the analyst should ensure that any tools are validated and appropriate for use in the context in which the intervention is to be used. The user-experience impact may be a fundamental contributor to the observed health impact or health system impact. For example, a recent DHI that utilized a personalized digital counseling application to enable consumer choice in contraceptive use found a positive effect on increasing adoption [35]. Economic evaluation that specifically identifies user experience (whether that user is a patient, the general population, a health provider or a health services manager) will be essential to accurate representation of the overall intervention impact.

The cross-sectoral impact enables a wider societal impact of a DHI to be incorporated, including impacts on other social sectors such as education. While the health impact, health system impact and cross-sectoral impact components of value representation are methodologically similar whether the intervention is digital or nondigital, the value of data and user-experience impact value categories are more specifically tailored towards DHIs. It is likely that there may not be quantitative information for some aspects of the impact inventory, in which case a qualitative description of the impacts should be provided.

The value-of-data component requires explicit identification of what evidence is being generated by the DHI. This could range from simple utilization data to comprehensive datasets on clinical pathways, processes and patient outcomes. This value is likely to be contingent on the context of the DHI and in particular the existing information systems and digital infrastructure, further highlighting the need for explicit specification of context in Step 1.

### Value aggregation

A central consideration in an economic evaluation is what, if any, aggregation of the costs and consequences is required and is appropriate.

Representing an incremental cost-effectiveness ratio with a generalized measure of health such as the quality-adjusted life year is commonly used for marginal health budget decisions under an extra-welfarist framework, where improvement in population health is a major, but not only, consideration of decision makers.

Estimating a return on investment (ROI), benefit–cost ratio or net benefits utilizing a benefit–cost analysis (BCA) analytical approach is commonly applied to major policy or regulatory changes or large-scale investments where there are multiple impacts across a range of constraints and sectors and the objective is to represent the impact and distribution on societal welfare. Health impacts of interventions can be monetized utilizing concepts such as the value of statistical life informed by valuation of mortality- or morbidity-risk reduction. Converting all positive and negative impacts into monetary form enables aggregation using a common metric, which enables clear and simple communication of results. Applying a BCA framework to DHI investments will require consideration of how the value categories introduced in the impact inventory can be monetized. Methodology for monetization of health value, processes value and cross-sector value impacts are largely in harmony with established BCA methods. If a total monetized summary metric is required in the analysis, the bespoke approaches would need to be adopted for monetization of evidence generation value, prediction value and disruption potential, with careful consideration required to ensure correct attribution and avoidance of double counting. Some emerging approaches that may be applicable for monetization of DHI-specific impacts are detailed in Table 5, and it is expected that ongoing methods research will generate more tailored methods.

**Table 5:** Approaches for monetization of DHI-specific impacts

**COMPONENT**	**ESTABLISHED/EMERGING APPROACHES TO MONETIZATION**
Health value	Value of statistical life (Robinson et al., 2019) [36]
Process value	Efficiency analysis (Jacobs et al., 2006) [37] Time preference (Whittington and Cook, 2019) [38] Contingent Valuation (Bayoumi, 2004) [39]
Cross-sectorial value	Economy-wide effects (Strzepek et al., 2018) [40]
Evidence-generation value	Data Shapley (Ghorbani and Zou, 2019) [41] Value of data assets (First San Francisco) [42]
Prediction value	Scenario analysis—preliminary development (Agrawal et al., 2019) [43]
Disruption potential value	Analysis of potential future scenarios with valuation

A social ROI analysis is a form of BCA. Building on the approach for estimating ROI, it explicitly attempts to identify and isolate wider social benefits from immediate financial returns of an investment and is useful where there are particular benefits that are considered socially desirable by a decision maker and that should be explicitly represented. The major types of value aggregation are detailed in Table 6.

**Table 6:** Key features of different types of value aggregation

TYPE OF VALUE AGGREGATION	REPRESENTATION	INCORPORATES	INTERPRETATION REQUIRES	FURTHER METHODOLOGICAL GUIDANCE
**Benefit–cost analysis**	Benefit–cost ratio: net benefits, return on investment	Full costs and benefits converted to monetized form to extent possible	Understanding of valuation technique, particularly health benefits	Benefit–cost analysis reference case [30]
**Cost utility analysis (a subset of cost-effectiveness analysis)**	Incremental cost effectiveness ratio (effect in generalized units) net health/monetary benefits	Costs and effects from a defined perspective	Marginal productivity of health system (k); ICERs of competing investments	Drummond et al. (2015) [1]; iDSI Reference case [31]
**Cost-effectiveness analysis**	Incremental cost effectiveness ratio	Costs and effects from a defined perspective, effects in natural units	ICERs of competing investments (in same effect units)	Drummond et al (2015) [1]
**Social return on investment**	SROI	All costs and benefits converted to monetized form, plus wider social impact	Understanding of relative weight or importance of additional social benefits	Olsen et al. (2003) [44]

*Note*. ICER = incremental cost-effectiveness ratio; iDSI = International Decision Support Initiative; SROI = social return on investment.

The complexity and variety of DHIs and wide range of potential decision makers necessitates that the concept of ‘return on digital investment’ utilizing the DHI economic evaluation framework does not prescribe a particular form of aggregation, but it does require that researchers and decision makers are cognizant of the options available, the additional information required for their interpretation and the risk of creating false certainty through obfuscation of important gaps in evidence, attribution and valuation.

The use of the impact inventory in addition to value aggregation will assist in transparent representation of results, and it is expected that a comprehensive assessment of the return on digital investment will involve a series of quantitative and descriptive outputs.

## IMPLICATIONS FOR RESEARCH AND POLICY

Any methodological guidance is only as good as the extent to which it improves the information available to decision makers. As a global public good, it is envisioned that payers and investors, product developers and researchers in developing countries will adopt and utilize this framework when planning, conducting and interpreting economic evaluations for DHIs. The framework is intended to integrate with existing guidance and is an initial step to address the observed gap in economic evaluation of DHI in developing counties. It builds on the principle-based concepts of the IDSI Reference Case for Economic Evaluation in developing countries [31], and the ongoing application of the framework will provide methodological consistency and further opportunity to refine the framework specifications. The framework has immediate application in supporting commissioned research and country technical assistance. The World Bank is utilizing the framework in two economic evaluations in the Ghanian health system and as part of digital health support to countries, further publications related to this work are forthcoming.

WHO’s *Guide to Monitoring and Evaluating DHIs* [12] notes the role of economic evaluation within the stages of the maturity life cycle of an intervention. Within the life cycle, an intervention can be at pre-prototype/protype, pilot, demonstration, scale-up or integration and sustainability phases. It is expected that this framework will be used to conduct economic evaluation predominantly at the demonstration and scale-up phases and following pilots. However, economic evaluation can be a useful tool for informing ongoing investment and modifications to established DHIs within the integration and sustainability phase.

The framework is not developed as a ‘rule book’ to constrain economic evaluation practice or create an unachievable standard to which economic evaluations of DHIs should be conducted. Nor is it a ‘textbook,’ seeking to replicate the extensive field of methodological practice in economic evaluation [31]. The developing country context results in specific challenges for DHI evaluation; the framework aims to acknowledge the uncertainty and variability that may be required for economic evaluation, while ensuring to core principles and standards are maintained. It is intended that the framework can be applied to evaluations conducted across a range of resources, time and evidence constraints. As the DHI landscape and evidence base is rapidly evolving, methodological innovation in the economic assessment of DHIs will be necessary, and it is expected that the framework will enable the development of a prioritized methodological research agenda in developing country contexts. The overarching concept when applying the framework is to ‘comply or justify’—i.e. users should attempt to adhere to the steps and principles of the framework, but where this is not feasible or appropriate, they should provide justification for the methodological choice [31]. In this way, the framework will facilitate methodological transparency and improve the overall usefulness of economic evaluations of DHIs.

## Data Availability

There are no new data associated with this article.
